# The Radiological Manifestations of the Aberrant Air Surrounding the Pleura: In the Embryological View

**DOI:** 10.1155/2012/290802

**Published:** 2012-04-10

**Authors:** Shih-Yi Lee, Chih-Hao Chen, Chin-Yin Sheu, Julie Hua Ying Tai, Sheng-Hsiung Yang, Chao-Hsien Chen

**Affiliations:** ^1^Division of Pulmonary and Critical Care Medicine, Mackay Memorial Hospital, Main Branch Hospital, Taipei 10449, Taiwan; ^2^Mackay Medicine, Nursing and Management College, Taipei 10449, Taiwan; ^3^Department of Thoracic Surgery, Mackay Memorial Hospital, Main Branch Hospital, Taipei 10449, Taiwan; ^4^Department of Diagnostic Radiology, Mackay Memorial Hospital, Main Branch Hospital, Taipei 10449, Taiwan; ^5^Semmelweis University, Budapest, Hungary; ^6^Division of Chest, Internal Medicine, Mackay Memorial Hospital, Main Branch Hospital, Taipei 10449, Taiwan

## Abstract

The radiological manifestations of the aberrant air surrounding the pleura are varied because of the air outlining the organs in and out of the visceral space. The continuity of the visceral space from the neck, mediastinum to the retroperitoneum is originated from embryological development, which is compatible with the findings through laboratory experiments, cadaveric anatomy, and thoracic computer tomography image. We reviewed the embryo development to understand the anatomy of body cavity, which can determine the radiological findings of pneumomediastinum and pneumothorax.

## 1. Introduction

**Figure 1:**
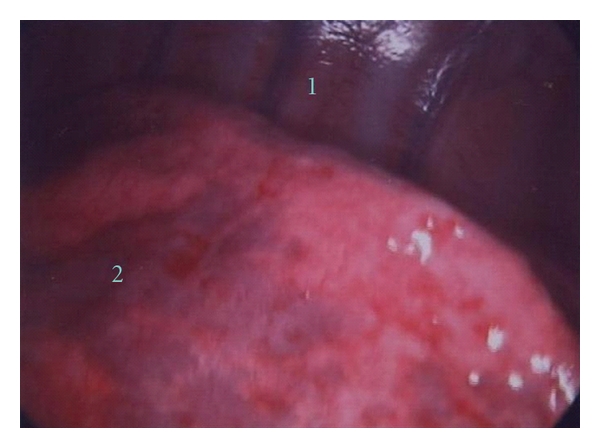
The thoracoscopic image shows the pleura overlaying the tissue and organs in the thoracic cavity: chest wall (1) and the lung (2).

During respiration, both lungs freely expand and collapse in the pleural space within the thoracic cavity. The pleural cavity, namely, is a space lined with pleura. The parietal pleura accompanied with ribs, muscles, and skin constitutes the thoracic wall. The visceral pleura covers the surface of both lungs (Figure 1). 

**Figure 2:**
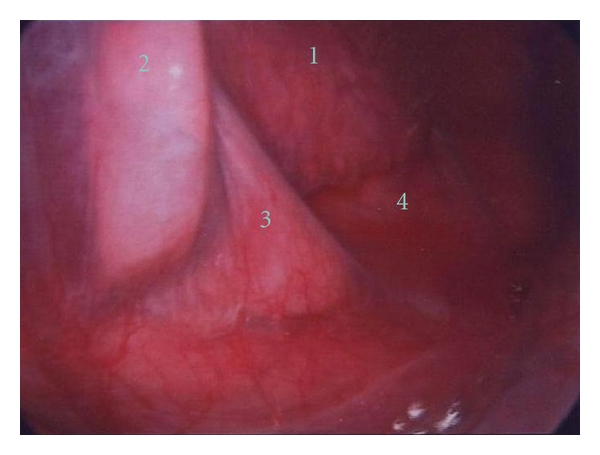
The thoracoscopic image shows the pleura overlaying the tissue and organs in the mediastinum: heart (1), descending aorta (2), inferior pulmonary ligament (3), and diaphragm (4).

 The visceral pleura overlies both lungs in addition to the organs in the mediastinum [1] (Figure 2). It encloses a space, which is known as visceral cavity. The visceral cavity is continuous from the neck to upper abdomen (the level of T2 to L1). These anatomical relationships are established as early as embryo development and greatly influence the radiological signs of aberrant air surrounding the pleura, pneumothorax, and pneumomediastinum [2–4].

Embryo development occurs in the period of 3rd to 8th week of gestational age. The three layers of the germ disc (ectoderm, mesoderm, and endoderm) gives rise to specific tissues and organs [5]. The following cephalocaudal and lateral foldings of the germ disc establishes the primal spatial relationships of the fetus among different tissues and organs, including thoracic and abdominal cages, pleura and peritoneum, trachea, and intestine.

In this review, we will focus on the formation of the continuum of visceral space during embryo development and its relationships to the radiological signs of aberrant air surrounding the pleura.

## 2. The Body Cavity Formation

After fertilization of the human ovum, the zygote procedes to several stages of development: morula, blastocyst, implantation, bilaminar germ disc, trilaminar germ disc, embryonic period, and fetal period.

**Figure 3:**
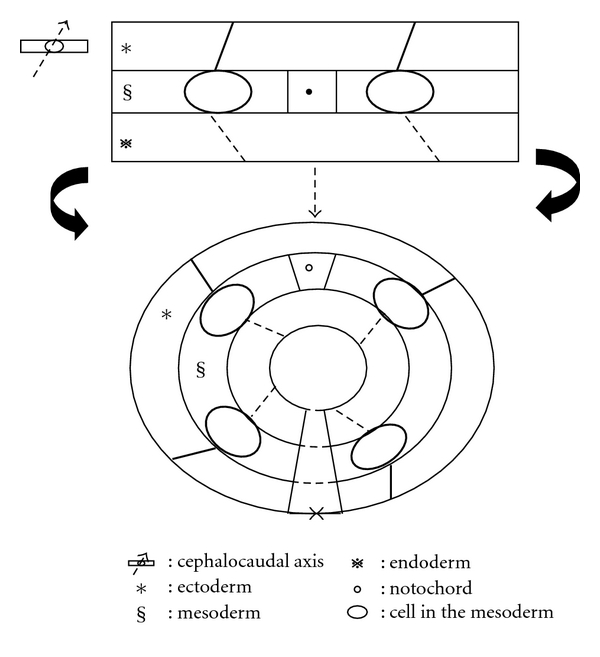
Diagram shows the model of lateral folding of germ disc.

**Figure 4:**
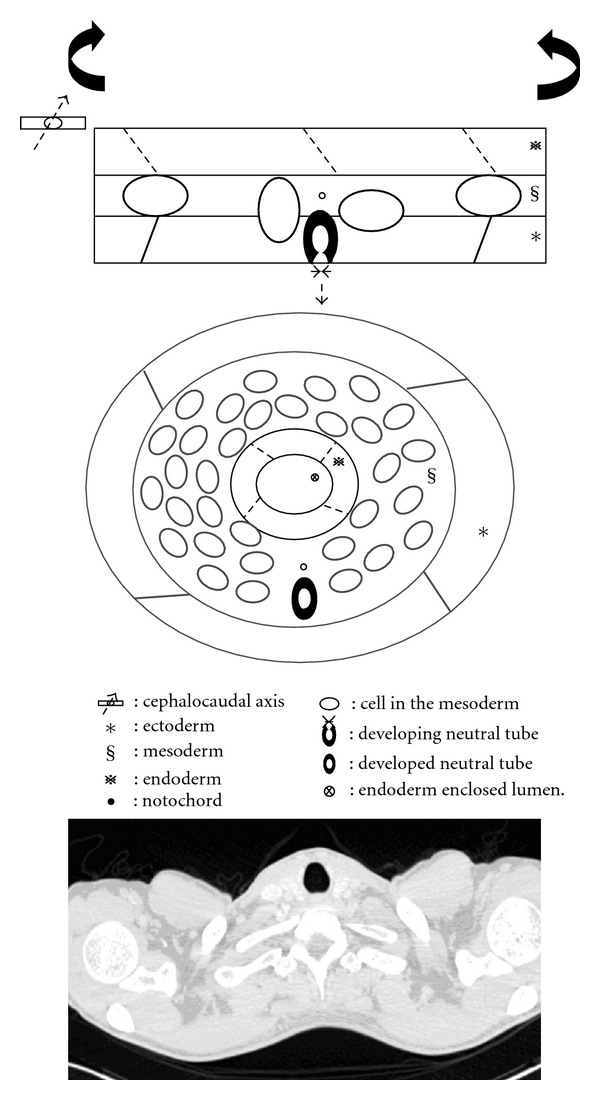
Lateral folding of germ disc in the level of pharyngeal arches. Diagram shows the cells in the mesoderm proliferates and neural tube is forming.

In the embryonic period, the lateral folding of the trilaminar germ disc forms the embryo in three-layer tube-like shape (Figure 3). This establishes the primitive anatomical relationships of the embryo: ectoderm on the surface, mesoderm in the middle, and endoderm inside. Nevertheless, this also shows the anatomical relationships of embryo on the level of pharyngeal arches [5] (Figure 4).

**Figure 5:**
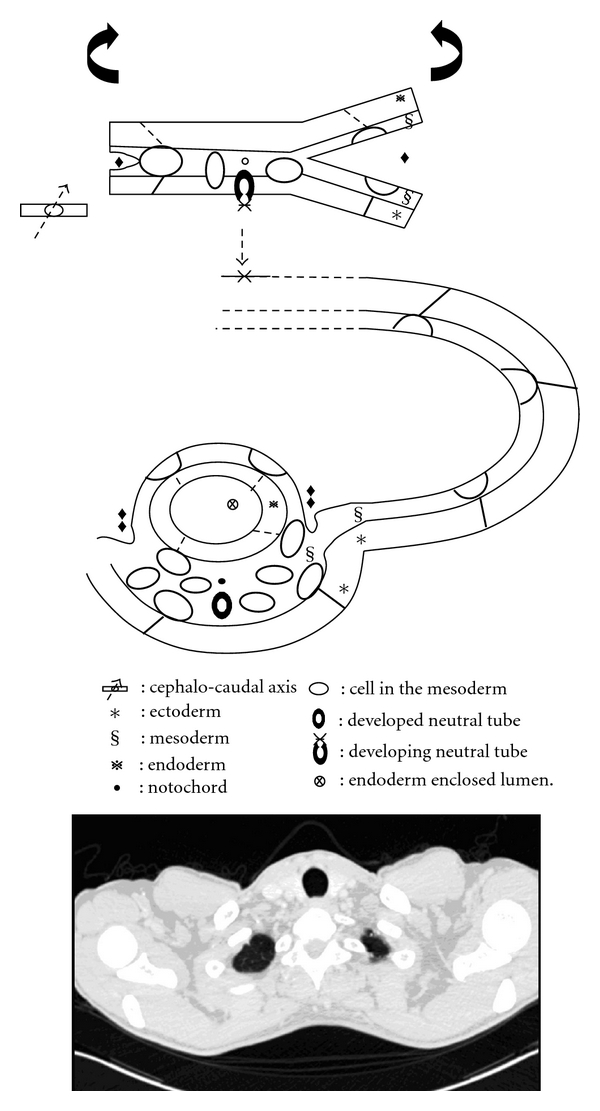
Body cavity formation. Diagram shows the mesoderm splits into parietal and visceral layer, which forms the intraembryonic coelomic cavity. The intraembryonic coelomic cavity is the primitive form of the body cavities.

The embryo below the level of pharyngeal arches develops to the body cavity, which is originated from the mesoderm on each side of the midline. The cells in the lateral plate of mesoderm starts to divide into the parietal and visceral mesoderms to form the intracellular cavities. The results of the lateral folding develops the parietal mesoderm and the ectoderm to form the body wall; the visceral mesoderm and endoderm to form the gut wall; the intracellular cavities within the mosoderm to form the intraembryonic coelomic cavity, which transforms to the body cavity afterward [5] (Figure 5).

## 3. The Compartmentalization of the Mediastinum and the Continuum of the Visceral Space of the Mediastinum

Although the body cavity is further divided into thoracic, abdominal, and pericardial cavities by the pleuroperitoneal and pleuropericaridial membranes, the cavities remain continuous throughout the visceral space [5]. 

**Figure 6:**
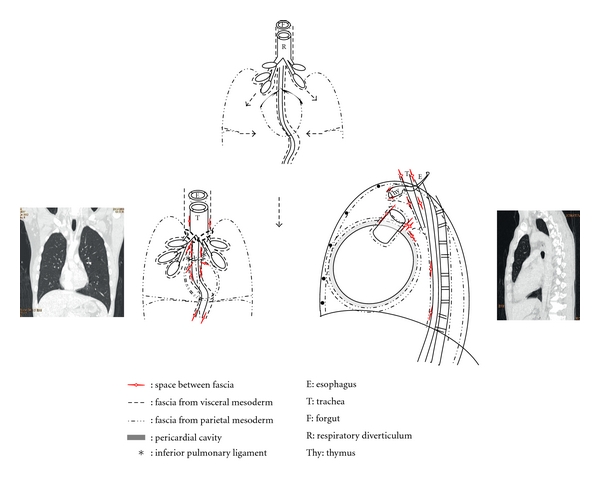
Diagram shows that the formation of the continuity of the fascial planes connects cervical soft tissue with the mediastinum to the retroperitoneum. The space between fascia permits aberrant air arising in any of these areas to spread elsewhere.

The visceral space around the esophagus, stomach, intestine, trachea, descending aorta, and azygos vein is called posterior mediastinum compartment, or post-vascular space [4, 6] (Figure 6). The visceral space from the neck to the upper abdomen, surrounded by the visceral fascia, is originated from the visceral mesoderm. The intraembryonic coelomic cavity of embryo is separated into the thoracic and abdominal cavities, after the pleuroperitoneal membrane and the transverse septum transforms into the diaphragm. The visceral facia in these two cavities, which is originated from the visceral mesoderm, becomes visceral pleura and peritoneum. The diaphragm does not hinder the continuity of the visceral mesoderm overlaying the gut. Therefore, the visceral space is continuous in the posterior mediastinum [4, 6] (Figure 6).

**Figure 7:**
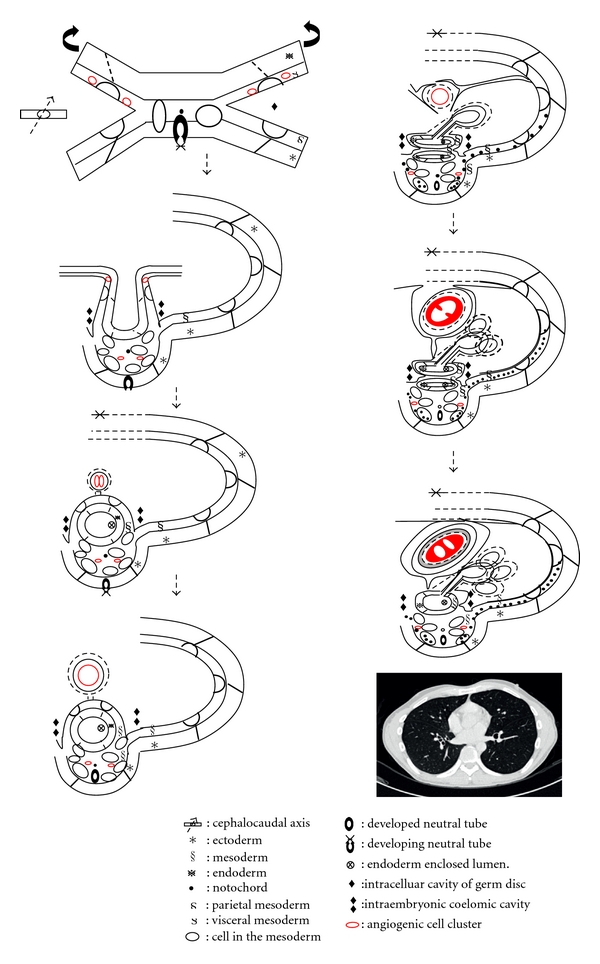
Heart and pericardial cavity formation. Diagram shows that bilateral angiogenic cell cluster originated from the mesoderm fused in the midline along with lateral folding of germ disc and forms the heart and the visceral pericardium. The parietal mesoderm then forms the fibrous and serous layer of parietal pericardium.

The space surrounding the pericardial and aortic fascia is known as middle mediastinum compartment or vascular space [7]. The formation of the pleuropericardial membrane of embryo separates to create a pericardial cavity within the thoracic cavity (Figure 7). However, the pericardial cavity and the post-vascular space of the thoracic cavity remains continuous by the vascular fascia (Figure 6). The angiogenic cell clusters, which are derived from the visceral mesoderm, transform into the cardiovascular system. These angiogenic cell clusters form the bilateral endocardial tubes. Accompanied with the cephalocaudal and lateral flexion of the germ disc, one end from each endocardial tubes fuse in the midline to develop the heart (Figures 7). The other end of the endocardial tubes unite in the midline of the embryonic shield to form the aortic arches. The aortic arches arise from the aortic sac and terminate at the dorsal aorta, forming lastly the arch of aorta. Since the aortic sac is the most distal part of the truncus arteriosus of the heart [5], the heart in the pericardial cavity is connected to the descending aorta through the aortic arch. Hence, the pericardial cavity and post-vascular space are continuous through the aortic fascia [6, 7] (Figure 7).

In the Zylak classification of mediastinum, the thoracic space anterior to the pericardium is the anterior mediastinum. Since the thymus originated from the third pharyngeal arch migrates caudally and medially into the anterior portion of the thoracic cavity [5], the upper portion of anterior mediastinum is continuous to the neck.

 The peribronchial and visceral spaces are continuous through each hilum. The lung bud is the respiratory primodrum, which is an outgrowth of the ventral wall of the foregut. The right lung bud is divided into three branches, while the left one is divided into two branches. It then extents caudally and laterally, and penetrates into the thoracic cavity [5] (Figure 6). Therefore, the hilum where the lung buds enter pleural cavity becomes the channel between lung interstitium and visceral space.

## 4. The Radiological Manifestations of Aberrant Air Surrounding the Pleura

**Figure 8:**
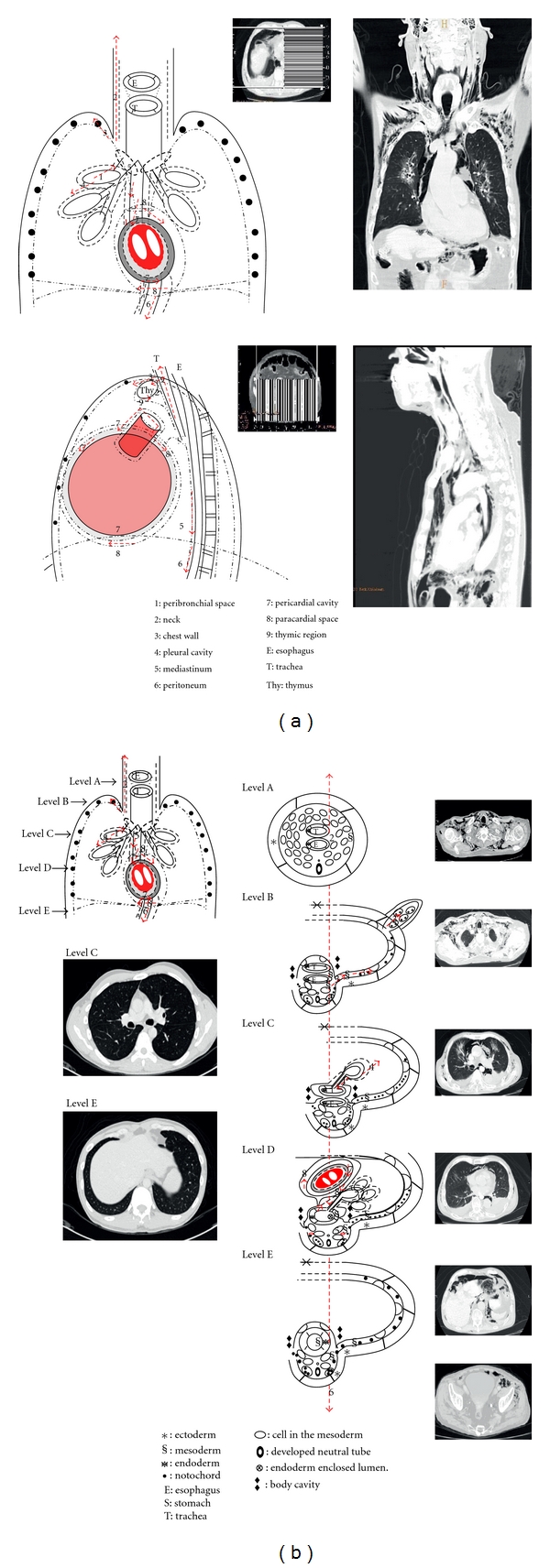
Air flow in the pneumomediastinum: peribronchial space (1); neck (2); chest wall (3); pleural cavity (4); mediastinum (5); retroperitoneum (6); pericardial cavity (7); paracardial space and diaphragm (8).

The embryonic development of compartmentalization of the mediastinum, and the continuum of the visceral space and its surrounding tissues, is associated well with the findings of the laboratory experiments [6, 8], cadaveric anatomy [7], thoracic computer tomography image [7], literature review on the clinical presentations [3, 9], and thoracic surgeon observations [6]. With excessive air leak from the normal lumen into the peribronchial space or visceral space, it can flow up to the neck and chest wall, down to retroperitoneum and thighs, anterior to thymus, even to peritoneum and pericardial space (Figures 8(a) and 8(b)). The air can leak from the vascular space further into the carotid sheath and the subcutaneous tissue (Figures 8(a) and 8(b)). Therefore, computed tomography image of the aberrant air in the visceral space can show pulmonary interstitial emphysema [10], pneumomediastinum [10], subcutaneous emphysema of neck, chest wall [11], or thigh, pneumopericardium [11], pneumoperitoneum [4], and pneumoretroperitoneum. The specific signs of pneumomediastinum, are caused by the normal structure outlined by the aberrant air. These include double bronchial wall sign, ring around the artery sign [3], aortic arch sign [3], continuous diaphragm sign [12, 13], extrapleural sign [14], and thymic sail sign [3, 15] (Figures 8(a) and 8(b)).

## 5. Conclusion

The embryo development establishes the anatomy of body cavity. The compartmentalization in the body cavity and the continuum of the visceral space has significant impact on the radiological manifestations of pneumomediastinum and pneumothorax. It is crucial to review the radiological manifestations of the aberrant air surrounding the pleura from the embryological view.
